# Diet Affects Muscle Quality and Growth Traits of Grass Carp (*Ctenopharyngodon idellus*): A Comparison Between Grass and Artificial Feed

**DOI:** 10.3389/fphys.2018.00283

**Published:** 2018-03-26

**Authors:** Honghao Zhao, Jianguo Xia, Xi Zhang, Xugang He, Li Li, Rong Tang, Wei Chi, Dapeng Li

**Affiliations:** ^1^Hubei Provincial Engineering Laboratory for Pond Aquaculture, National Demonstration Center for Experimental Aquaculture Education, College of Fisheries, Huazhong Agricultural University, Wuhan, China; ^2^Department of Animal Science, Institute of Parasitology, McGill University, Sainte-Ann-de-Bellevue, QC, Canada; ^3^Key Laboratory of Health Aquaculture and Product Processing, Hunan University of Arts and Science, Changde, China

**Keywords:** muscle growth and development, myogenic regulatory factors family (*MRFs*), muscle fiber, texture property, grass carp

## Abstract

Fish muscle, the main edible parts with high protein level and low fat level, is consumed worldwide. Diet contributes greatly to fish growth performance and muscle quality. In order to elucidate the correlation between diet and muscle quality, the same batch of juvenile grass carp (*Ctenopharyngodon idellus*) were divided into two groups and fed with either grass (*Lolium perenne, Euphrasia pectinata* and *Sorghum sudanense*) or artificial feed, respectively. However, the different two diets didn't result in significant differences in all the detected water quality parameters (e.g., Tm, pH, DO, NH_3_/${\text{NH}}_{4}^{+}$-N, ${\text{NO}}_{3}^{-}$-N, ${\text{NO}}_{2}^{-}$, TN, TP, and TOC) between the two experimental groups. After a 4-month culture period, various indexes and expression of myogenic regulatory factor (MRFs) and their related genes were tested. The weight gain of the fish fed with artificial feed (AFG) was nearly 40% higher than the fish fed with grass (GFG). Significantly higher alkaline phosphatase, total cholestrol, high density cholestrol and total protein were detected in GFG as compared to AFG. GFG also showed increased hardness, resilience and shear force in texture profile analysis, with significantly bigger and compact muscle fibers in histologic slices. The fat accumulation was most serious in the abdomen muscle of AFG. Additionally, the expression levels of *MyoG, MyoD, IGF*-*1*, and *MSTNs* were higher, whereas *Myf*-*5, MRF4*, and *IGF*-2 were lower in most positional muscles of GFG as compared to AFG. Overall, these results suggested that feeding grass could promote muscle growth and development by stimulating muscle fiber hypertrophy, as well as significantly enhance the expression of *CoL1A*s. Feeding *C. idellus* with grass could also improve flesh quality by improving muscle characteristics, enhancing the production of collagen, meanthile, reducing fat accumulation and moisture in muscle, but at the cost of a slower growth.

## Introduction

Fish muscle development and growth are complex dynamic processes involving both the recruitment of new muscle fibers (hyperplasia) and the growth of existing fibers (hypertrophy). Different from mammals, fish have a long-lasting ability to recruit new skeletal muscle fibers, persisting from larval life stage till juveniles reaching adult body size (Stickland, 1983, Weatherley et al., 1988; Rowlerson and Veggetti, 2001). Identification of the factors involved in regulating hyperplasia and hypertrophy in fish muscle has great potential to optimize muscle quality.

It's well-known that fish growth and flesh quality are affected by both external and internal factors, such as aquaculture models, rearing conditions, ration level, genetics, and so on (MacDonald and Webber, 1995; Johnston, 2001; Łuczyn et al., 2009; Yan et al., 2013; Gutierrez et al., 2014; Gisbert et al., 2016). Among them, food sources and nutrients are considered as primary contributors. In particular, the effects of diet nutrient levels on muscle fiber characteristics, including size and number of muscle fibers, intramuscular fat content, moisture and so on, have been widely reported no matter in mammals and fish species (Joo et al., 2013; Li et al., 2016). The changes in dietary protein level mainly resulted in a decrease in the muscle fiber size as the total number of fibers did not vary significantly in *Senegalese sole* (Luisa et al., 2016). Fish oil substitution by 66% vegetable oils has an effect on the adipocyte size in *Sparus aurata* L. (Cruz et al., 2011). Changes in dietary lipid sources also affected the number of white muscle fibers in *Oncorhynchus mykiss* (Fauconneau et al., 1997). Lysine supply caused decline in muscle fiber diameter and number when compared to the treatment with a commercial diet in nile tilapia (*Oreochromis niloticus*) (Aguiar et al., 2005). A recent study reported that muscle protein content and water holding capacity were significantly elevated, while moisture, lipid and ash contents were significantly decreased with increased dietary phosphorus level (up to 5.6 g/kg) in *C. idellus* (Wen et al., 2015).

Muscle quality is influenced by muscle fiber characteristics and other endogenous factors such as internal cross-linking of connective tissue, texture, fatty acid compositions, etc. (Cheng et al., 2014). Myotome is one of most important constituents of muscle. It is made up of a large number of single muscle fibers linked by intramuscular connective tissues. Collagen is the most significant constituent of the connective tissues. It plays a vital role in maintaining filet integrity and muscle cohesiveness (Bjørnevik et al., 2004; Aussanasuwannakul et al., 2012). Previous studies were focused mainly on the influence of different diets on muscle texture, without thoroughly investigating the link between muscle texture and its molecular regulation (Mommsen, 2001; Gong et al., 2017).

The growth of fish muscle is regulated by various genes. Among them, the growth hormone (GH)-insulin-like growth factor (IGF) system is the key regulator of growth in vertebrates. How this system modulates muscle mass in fish is just recently becoming elucidated (Fuentes et al., 2013). In fish, myogenesis involves the specific regulation of myogenic regulatory factors (MRFs), which control specification, activation, and differentiation of myogenic cells (Watabe, 1999). Muscle growth is also modulated by myostatin (MSTN), which normally inhibits the growth of skeletal muscle (McPherron et al., 1997). Both postnatal aquaculture environment and dietary factors can affect expression levels of MRFs and their related positive & negative regulating genes, as shown in *O. mykiss* (Alami et al., 2010), *Piaractus mesopotamicus* (Gutierrez et al., 2014), *O. niloticus* (Nebo et al., 2013), and *C. idellus* (Lin et al., 2015; Zheng et al., 2015). Despite the increasing researches on the relationships between MRFs and their target genes, a comprehensive study on *C. idellus* is still lacking.

*Ctenopharyngodon idellus* is the principal species in freshwater aquaculture in China (He et al., 2015). Intensive farming based on utilization of artificial formulated feed has contributed to the continuous growth in the production of grass carp (Borlongan and Satoh, 2002; FAO Yearbook, 2014). Unfortunately, the flesh quality of farmed grass carp declined over time through the course of artificial aquaculture and has led to increasing public concerns (Qin, 2010). To get high quality fish products is a key target for successful aquaculture industry in the future (Li et al., 2013; Valente et al., 2013). Given that diet is a key contributor to fish flesh quality, it is important to assess whether feeding natural grass could improve the flesh quality of *C. idellus*. To this aim, we have conducted a comprehensive study to compare the effects of natural grass and artificial feed on fish muscle characteristics, and explored the link between fish culture modes and flesh quality. To help elucidate the underlying molecular mechanism, we further investigated the expression of important genes between different feeds. The result could provide a theoretical basis and reference for further research.

## Materials and methods

### Experimental design

The experiment was carried out in filed culture ponds in Chonghu Fish Farm, Hubei Province, China. Water temperature was between 22°C in July, 26°C in August–October. Despite of the Tm, the other water quality parameters of each culturing pond were monitored in the middle of each month, meanwhile, 2 days before sampling, the physical and chemical parameters of the water in temporary tanks were also detected and analyzed. *C. idellus* used in this study were the same batch of cultivated *C. idellus* fingerling with average initial weight at 35 g per tail. These fish were divided into two groups with three replicate ponds for each group. At the beginning of the experiment, about 3,000 tails of *C. idellus*, polycultured with 550 tails of *Hypophthalmichthys molitrix* (about 14 g per tail) and 350 tails of *Aristichthys nobilis* (about 25 g per tail), were assigned to each pond (the area is 22666.67 m^2^/pond). Fish in the grass feed group (GFG) were fed with 100 kg grass per pond, at 9 am per day, they include *Lolium perenne, Euphrasia pectinata* and *Sorghum sudanense* (crude protein 15.30%, crude fat 2.88%, moisture 78.40%, crude fiber 25.90%, ash 3.50%). The natural grass were planted along the culture ponds of both experimental groups, which could provide adequate food sources for the fish of GFG. Whereas, the fish in the artificial feed group (AFG) were fed with commercial feed (Haid, China), which contain crude protein 28.00%, crude fat 7.06%, moisture 8.75%, crude fiber 15.00%, and ash 15.63%. 15 kg artificial feed were supplied to each pond of AFG at 9 am and 15 pm per day. All the farmed *C. idellus* were fed to satiation during the whole rearing period. This experiment was carried out from 8th, July to 28th, October, 2016.

At the end of this experiment, 150 fish in each group were captured and then transferred to the laboratory in the College of Fisheries, Huazhong Agricultural University. Finally, 120 fish in each group were used for growth performances data measure and samples collection.

The Animal Research: Reporting of *In Vivo* Experiments (ARRIVE) guidelines and “Guidelines for Experimental Animals” of the Ministry of Science and Technology (Beijing, China) were compiled during the experimental period. Institutional Animal Care and Use Ethics Committee of Huazhong Agricultural University had approved our study. All efforts were made to minimize suffering of sampled fish species.

### Pond water quality measurement

During the 4-month culture period, the rearing conditions and water quality parameters were monitored and analyzed timely. Water temperature (Tm) of the two experimental groups ponds were both between 22°C in July, 26°C in August–October. Despite of the Tm, the other water quality parameters of each culturing pond, such as pH, dissolved oxygen (DO), ammonia nitrogen (NH_3_/${\text{NH}}_{4}^{+}$-N), nitrate nitrogen (${\text{NO}}_{3}^{-}$-N), nitrite nitrogen (${\text{NO}}_{2}^{-}$-N), total nitrogen (TN), total phosphorus (TP) and total organic carbon (TOC), were also measured in the middle of each month. Meanwhile, 2 days before sampling, the physical and chemical parameters of the water in temporary tanks were also detected and analyzed.

Among these tested water quality parameters, Tm, pH, and DO values were directly detected by Multi-Parameter Water Quality Detector. The TOC values were tested and analyzed by nondispersive infrared absorption method (GB/T13193-1991). Otherwise, the TN and TP were detected according to alkaline potassium persulphate digestion-UV spectrophotometric method (GB/T11894-1989) and ammonium molybdate spectrophotometric method (GB/T 11893-1989), respectively. The values of NH_3_/${\text{NH}}_{4}^{+}$-N, ${\text{NO}}_{3}^{-}$-N, and ${\text{NO}}_{2}^{-}$-N were also measured and calculated by their corresponding national standard methods: GB/T7479-1987, GB/T 7480-1987, and GB 7493-1987.

### Measurement of growth traits

Upon arrival, *C. idellus* of the two experimental groups were respectively stocked in temporary rearing tanks for 1 week. The feeding was stopped 2 days before sampling. The sampled fish was anesthetized in 100 mg·L^−1^ tricaine methanesulfonate (MS-222, Sigma, St. Louis, Missouri, USA) before measuring the growth indexes and samples collection.

The body weight, body length and body height of total 120 tails fish per group were measured. After collecting blood samples, the visceral mass cut off from esophagus to cloacal orifice, were took out from abdominal cavity and weighed. Then, the liver tissues were totally separated from the visceral mass and weighed the fresh weight again. Afterwards, the specific growth rate (SGR) and condition factor (CF) were calculated based on the measured data.

$$\begin{array}{l} \text{S}\text{G}\text{R}=\left(\text{Ln}\left(\text{W}1\right)-\text{Ln}\left(\text{W}2\right)\right)/\text{T}{\;}^{*}\;100\\ \text{ C}\text{F}=\left(\text{W}1/{\text{L}}^{3}\right){\;}^{*}\;100 \end{array}$$

Note: W1-Body Mass (g); W2-Initial Weight (g); T-Feeding days; L-Body Length (cm).

### Samples collection

#### Blood sampling

The blood samples (180–200 mL per tail) from 10 fish each group were taken from caudal vein without an anti-coagulating substance by injector puncture. The blood samples were placed at room temperature for 30 min, then centrifuged at 3,000 g for 30 min at ordinary temperature for serum preparation. The separated serum was stored at −80°C until the serum biochemical indexes analysis.

#### Muscle tissue sampling

Histological section was used to analyze the characteristic of muscle fiber Four positional muscle samples were respectively collected from back, rib, abdomen, and tail parts. The fresh biopsy specimens (transversely larger than 5 × 5 mm at minimum; vertically larger than 5 × 8 mm at minimum) were preserved in 4% paraformaldehyde at room temperature.

**Figure 1:**
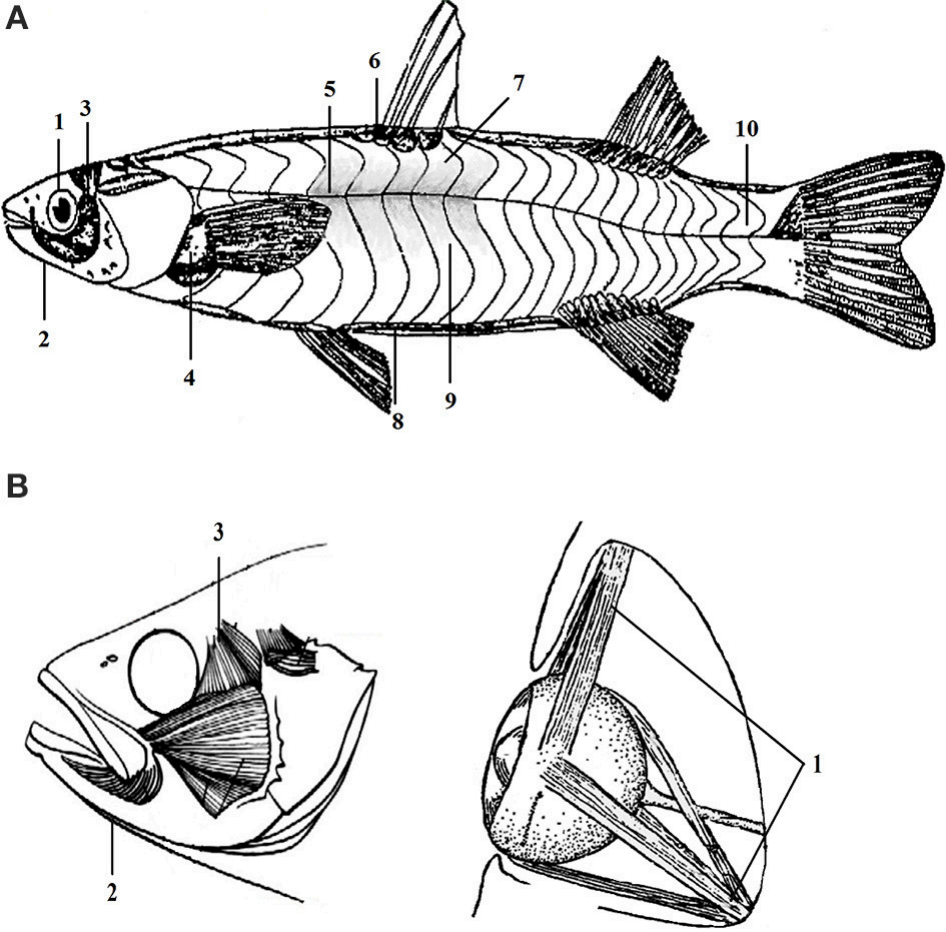
The distribution of different positional muscle tissues of *C. idellus*. The different positional muscle samples tested in this study were collected from corresponding designated parts of *C. idellus*. General profile of the distribution of 10 positional muscles was shown in section **(A)**. Detailed distribution of muscles in head was described in section **(B)**. The part of fish body in light gray, is given only as a spatial reference **(A)**. Corresponded to the muscle analyzed in present study, the gray part is superficial red muscle. The 10 numbers in **(A)** represent 10 positional muscles. 1-eyes muscle; 2-jaw muscle; 3-opercular muscle; 4-paired fins muscle; 5-red muscle; 6-odd fin muscle; 7-back muscle; 8-abdomen muscle; 9-rib muscle; 10-tail muscle.

The expression levels of multi-gene were detected in 10 positional muscle tissues, i.e., back, rib, abdomen, red, tail, odd fin, paired fins, opercula, jaw and eyes muscles. The 10 tested muscle tissues were collected based on the general profile of the distribution of 10 positional muscles (Figure 1). This sampling standard was rigorously followed during the experiments.

### Serum biochemical assay

Serum samples were prepared according to the previous method (Shi et al., 2006). The lactate dehydrogenase (LD), alkaline phosphatase (ALP), total cholesterol (TCHO), high density cholesterol (HDLC), glucose (GLU), albumin (ALB), total protein (TP), and triglycerides (TGK) were tested by automatic biochemistry analyzer (Hitachi 7020, Hitachi High Technologies, Inc., Ibaraki, Japan). Test kits were purchased from Nanjing Jiancheng Biochemical Corporation (Nanjing Jiancheng Biochemical Corporation, Nanjing, China), and the procedures followed were in accordance with the instructions of the kit.

### Muscle texture measurements

Muscle texture assay was performed according to the method described by Ma et al. (2014). Muscle above the lateral line was dissected from 24 fish for each culture model, and then was cut into 1 × 1 × 1 cm block for TPA test and 1 × 1 × 4 cm for shear force test. The Texture Analyser used is TA-XT Plus Micro TPA (Stable Micro Systems, Surrey, England) equipped with a flat bottomed cylindrical probe P/36R. The type of loading probe is Auto-5 g, and the samples were compressed for 2 times in TPA test. Test condition was recorded at a pre-test speed of 3 mm/s, a test speed of 2 mm/s and a post-test speed of 2 mm/s with the stay time at 5 s, the data collection rate was 200 pps. Filet heights and maximum force (g shear force) needed to compress filets 65% of their height. 65% was chosen together with rounded probe ends as to reduce damage of muscle structures (Hyldig and Nielsen, 2001). The samples of TPA and shear force tests were carried out at room temperature, with 10 fish in each culture model and five parallel samples were taken in TPA test. Three measurements were performed on each filet in shear force test (1 cm between each measure point) centrally on the flesh side and the thickest muscle layer. Texture curves were recorded and the max force was determined as an average of the three measurements.

### Histological analysis

Serial transverse 10 μm-thick sections were stained with hematoxylin & eosin (H & E staining) and intracytoplasmic lipids with oil red O (Oil O staining) according to the procedures (Rasmussen and Ostenfeld, 2000), respectively.

A total of 200–400 fibers of white muscle per fish were studied in a Leica MZ 6 microscope for their cross sectional area (CSA) and the diameter (*d* = 2r) of each fiber was calculated from the fiber area (A) [A = π·r^2^), thus, d = 2√ (A·π^−1^)]. A size limit for identifying fibers was set at fiber diameters ≥10 μm since the optical resolution below this limit did not allow for sufficient identification and accuracy in the analyses (Luther et al., 1995). The circularity of each fiber (4π·fiber area·circumference^−2^) was also determined. A circularity of 1.0 indicated a complete circle. The free software Image J (http://rsb.info.nih.gov/ij/) was used for quantitative statistics and analyses.

### Molecular cloning and analysis

The tissues were homogenized by grinding apparatus to extract total RNA using RNAiso reagent (TaKaRa, Shuzo, Japan) according to the manufacturer's instructions. The total RNA was then dissolved in 50 μL RNase-free water. The quality of total RNA was checked with the agarose gel and concentration were measured by NanoDrop 2000 (Thermo Scientific, Waltham, MA, USA). The PrimeScript RT reagent Kit with gDNA Eraser (TaKaRa, Shuzo, Japan) was used to reverse-transcribe the first strand cDNA from the total RNA. The partial *C. idellus* cDNA sequences were obtained from National Center for Biotechnology Information (NCBI^1^), which include *MRF*s (myogenin, *MyoG*; myogenic differentiation, *MyoD*; myofactar-5, *Myf*-*5*; myofactar-6, *MRF4*); myostatin type I and type II (*MSTN*-*1*/*MSTN*-*2*); insulin-like growth factor 1 (*IGF*-*1*); insulin-like growth factor 2 (*IGF*-*2*); collagen type I alpha-1 (*COL1A*-*1*) and collagen type I alpha-2 (*COL1A*-*2*). Special amplified primers of these genes cDNA were given in **Table 3**. PCR products were gel-purified, ligated into the T/A cloning vector pMD-19T (Takara, Dalian, China) and transformed into *Escherichia coli* DH5α competent cells. Positive clones were examined by PCR followed by direct sequencing.

### Quantitative real-time PCR analysis

Expression patterns of these genes (*MyoG, MyoD, Myf*-*5, MRF4, MSTN*-*1, MSTN*-*2, IGF*-*1, IGF-2, COL1A*-*1*, and *COL1A*-*2*) were analyzed using quantitative real-time PCR (qRT-PCR). The β-*actin* and *18s* rRNA were selected as reference genes. All primer sequences were described in Table 1. The qRT-PCR assay was performed using SYBR Premix Ex Taq™ (TaKaRa, Dalian, China) on a Roche Light Cycler 480 machine (Roche, Sussex, UK). The qRT-PCR conditions were as follows: denaturation at 95°C for 30 s, followed by 40 cycles of 95°C for 5 s, annealing at 58°C (*MyoG*)/60°C (*MyoD*)/60°C (*Myf*-*5*)/56°C (*MRF4*)/58°C (*MSTN*-*1*)/58°C (*MSTN*-*2*)/60°C (*IGF*-*1*)/58°C (*IGF*-*2*)/55°C (*COL1A*-*1*), and 55°C (*COL1A*-*2*) for 20 s, and elongation at 72°C for 15 s. The relative quantification of the target and reference genes was evaluated according to standard curves. Each experiment was conducted in triplicate. The relative stability measures (M) of the reference genes were calculated by GeNorm^2^ (http://medgen.ugent.be/genorm/). Based on the results, β-*actin* was chosen as the reference gene in subsequent analyses.

**Table 1:** The primer sequences for genes cloning and expression in the study.

**Target gene**	**GeneBank no**.		**Primer sequence (5′-3′)**
**INTERNAL CONTROL PRIMERS FOR qRT-PCR**			
β-*actin*	DQ211096	Sense	CAGAGCTTCTCCTTGATGTC
		Antisense	GATATGGAGAAGATCTGGC
*18s*	EU047719	Sense	GGCGCGCAAATTACCCATTT
		Antisense	TCCCGAGATCCAACTACAAGC
**QUANTITATIVE PRIMERS OF THE GENES**			
*MyoG*	JQ793897	Sense	AGAGGAGGTTGAAGAAGGTC
		Antisense	GTTCCTGCTGGTTGAGAGA
*MyoD*	GU218462	Sense	CCCTTGCTTCAACACCAACG
		Antisense	TCTCCTCTCCCTCATGGTGG
*Myf*-*5*	GU290227	Sense	GGAGAGCCGCCACTATGA
		Antisense	GCAGTCAACCATGCTTTCAG
*MRF4*	KT899334	Sense	TCATTCAACTTGTGCCCTCC
		Antisense	GCCCACTTTGCGATACCC
*MSTN*-*1*	KM874826	Sense	GCAGGAGTCACGTCT TGGCA
		Antisense	GAGTCCCTCCGGATTCGCTT
*MSTN*-*2*	KM874827	Sense	GACCACA CCAGGGTCCAAC
		Antisense	TCACACTGGTAAAGCCCGTCAA
*IGF*-*1*	EU051323	Sense	GCTGCAGTTTGTGTGTGGAG
		Antisense	ATGCGATAGTTTCTGCCCCC
*IGF*-*2*	EF062860	Sense	GCTTCCACAAACCGCCTACC
		Antisense	AAAGAGTCTCCGCCGTTGCT
*COL1A*-*1*	HM363526	Sense	ACGCACACAAACAATCTCAAGT
		Antisense	GCATGGGGCAAGACAGTCA
*COL1A*-*2*	HM587241	Sense	ACTCCGATAGAGCCCAGCTT
		Antisense	ACATTGGTGGCGCAGATCA

### Statistical analysis

The white muscle fiber circularity data were ln-transformed by software Image J. Fiber diameter was additionally fitted as a covariate to fiber circularity. For comparison of the distributions of muscle fiber sizes, the non-parametric Kolmogorov-Smirnov two sample test was applied. This test is very sensitive in detecting differences in dispersions and skewness compared to other statistical tests (Zar, 1996). One-way ANOVA was used for analysis of differences in the two frequencies of each fiber diameter category. Differences were considered significant at *P* < 0.05 and highly significant at *P* < 0.01.

For statistical analysis, data from qRT-PCR were all presented as the mean ± S.E. for each genes (*MRF*s, *MSTN*-*1, MSTN*-*2, IGF*-*1, IGF*-*2, COL1A*-*1*, and *COL1A*-*2*). The optimized comparative Ct (2^−ΔΔCT^) value was used to compute the relative expression level (Livak and Schmittgen, 2001). One-way ANOVA and *t*-test were used to compare the significance of genes among different positional muscle samples and the expression of each gene in the same muscle position between the two culture models *C. idellus*. Meanwhile, Duncan's test was also applied to multiple comparisons by IBM SPSS Statistics 19.0 (SPSS, Chicago, IL, USA).

The other analyses of variance were performed either on means of triplicates (growth performances, blood biochemical indexes and texture indexes). The 0.05 probability level was used to denote data that were statistically significant, while 0.01 probability level was used to denote data that were highly significant.

## Results

### Water quality parameters

**Figure 2:**
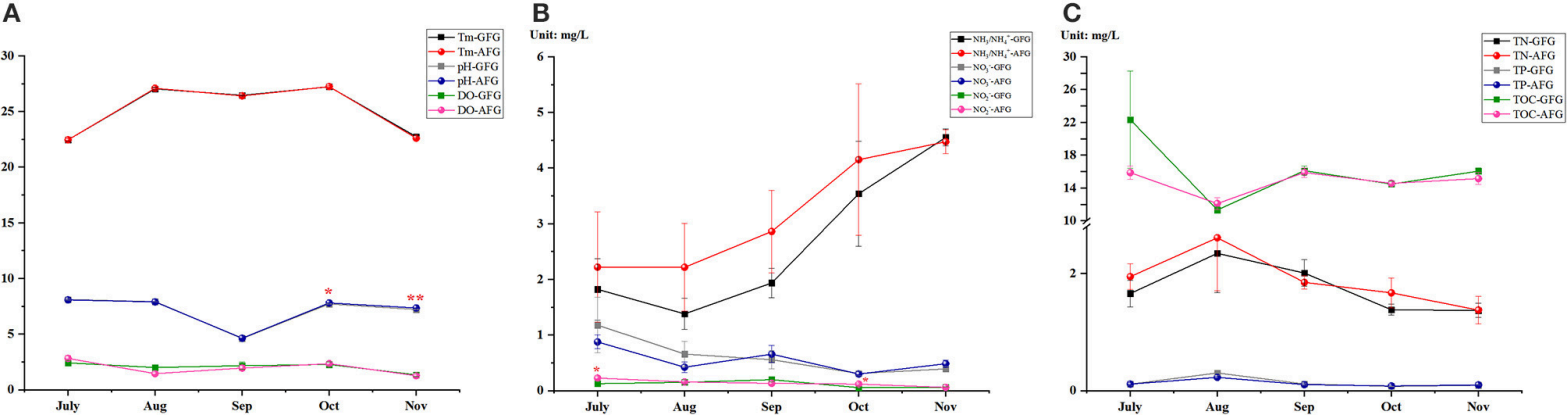
Water quality parameters of two experimental groups. The different chemical elements in water body of grass feed group (GFG) and artificial feed group (AFG), including **(A)** temperature (Tm), dissolved oxygen (DO), pH; **(B)** various forms of nitrogen (NH_3_/${\text{NH}}_{4}^{+}$, ${\text{NO}}_{3}^{-}$, ${\text{NO}}_{2}^{-}$) and **(C)** the total amount of various chemical elements (total nitrogen—TN, total phosphorus—TP, and total organic carbon—TOC) were tested in every month from July to November. Vertical bars represent the mean ± S.E. Meanwhile, the red color star bars indicate the significance between the two experimental groups, ^*^means significant (*P* < 0.05), ^**^means greatly significant (*P* < 0.01).

During the 4-month breeding cycle, the results of water quality parameters were showed Figure 2. The results of pH were higher in AFG since September, in especial, significances were tested in October and November (*P* < 0.05). Most of the DO values were higher in the GFG with no significance (Figure 2A). Among the detections of various forms of nitrogen, most of the measured NH_3_/${\text{NH}}_{4}^{+}$-N and ${\text{NO}}_{2}^{-}$-N values were higher in AGF, particularly, the detected concentrations of ${\text{NO}}_{2}^{-}$-N in July and October were significantly higer in AFG (*P* < 0.05). In addition, as time goes on, the concentrations of NH_3_/${\text{NH}}_{4}^{+}$-N increased in both groups, however, the change trend of ${\text{NO}}_{3}^{-}$-N concentrations were opposite. The other aspect, no significance was found in all the test results of TN, TP, and TOC measurement (Figure 2C). In general, no matter which test indicator, their variation tendency in GFG were consistent with the change trendency of the corresponding test indicator in AFG.

### Growth performance

Table 2 shows the growth performance of the two experimental groups *C. idellus* fed with artificial feed and grass feed, respectively. The average initial weight of the two groups fish was about 35 g per tail. After 113 days of separate feeding, the body mass, body length, body height, visceral weight and liver weight, even SGR were all significantly higher in AFG (*P* < 0.01). Only the CF was significant higher in GFG (*P* < 0.05).

**Table 2:** Growth performance of *C. idellus* fed with different feeds.

**Experimental group**	**Body mass**	**SGR**	**Body length**	**Body height**	**Visceral weight**	**Liver weight**	**CF**
Unit	g	%	cm	cm	g	g	%
Grass feed	993.77 ± 41.66	3.26 ± 0.02	31.37 ± 0.24	6.50 ± 0.12	36.23 ± 1.19	7.19 ± 0.18	3.32 ± 0.03
Artificial feed	1386.27 ± 37.31^**^	2.96 ± 0.04^**^	34.52 ± 0.18^**^	7.25 ± 0.08^**^	55.70 ± 2.33^**^	13.99 ± 0.12^**^	3.21 ± 0.00^*^

The data-measured traits of growth performances were represented by mean ± SE;

** difference between the two experimental groups is significant at the 0.01 level;

* *difference is significant at the 0.05 level*.

### Serum biochemical parameters

Table 3 shows the serum biochemical data of *C. idellus* in the two experimental groups. The comparison results indicate that different feeding diet had significant effect on LD, ALP, TCHO, HDLC, and TP concentrations (*P* < 0.05). The great significances were observed in the concentrations of LD between the two culture models (*P* < 0.01). No significant differences were observed in the concentrations of GLU, ALB, and TGK between GFG and AFG.

**Table 3:** Serum biochemical parameters in *C. idellus* cultured under two feeding models.

**Indicator**	**LD**	**ALP**	**TCHO**	**HDLC**	**GLU**	**ALB**	**TP**	**TGK**
Unit	U/L	U/L	mmol/l	mmol/l	mmol/l	g/l	g/l	mmol/l
Grass feed	829.17 ± 6.87^**^	132.83 ± 5.09^*^	7.69 ± 0.02^*^	2.50 ± 0.03^*^	3.68 ± 0.07	3.33 ± 0.33	36.50 ± 0.58^*^	6.74 ± 0.14
Artificial feed	454.58 ± 9.20	109.58 ± 2.76	6.70 ± 0.25	2.21 ± 0.06	2.96 ± 0.33	3.67 ± 0.30	31.17 ± 1.17	7.09 ± 0.13

*Asterisks expressed the significant difference*.

* Significance is at the 0.05 level;

** *significance is at the 0.01 level*.

### Muscle texture profiles

The data of texture measurements are represented in Table 4. Significant differences were detected in adhesiveness, springiness, cohesiveness, gumminess, chewiness, resilience, and shear force between the two groups (*P* < 0.05) except the hardness. Adhesiveness, springiness, cohesiveness, gumminess, and chewiness were significantly higher in AFG (*P* < 0.05), while resilience and shear force were significantly higher in GFG (*P* < 0.01).

**Table 4:** Comparison of texture measurements between two feeding model *C. idellus*.

**Group**	**Hardness**	**Adhesiveness**	**Springiness**	**Cohesiveness**	**Gumminess**	**Chewiness**	**Resilience**	**Shear force**
Grass feed	5318.43 ± 22.76	−11.69 ± 0.37^**^	0.54 ± 0.02^*^	0.26 ± 0.00^**^	1405.40 ± 68.16^**^	755.11 ± 27.18^**^	0.16 ± 0.00^**^	503.11 ± 3.89^**^
Artificial feed	5294.80 ± 54.59	−25.92 ± 2.20	0.60 ± 0.00	0.29 ± 0.00	1520.71 ± 75.16	909.20 ± 43.56	0.13 ± 0.00	360.62 ± 1.96

The data of measured traits were expressed by mean ± SE;

** difference between the two experimental groups is significant at the 0.01 level;

* *difference is significant at the 0.05 level*.

### Histological observation

**Figure 3:**
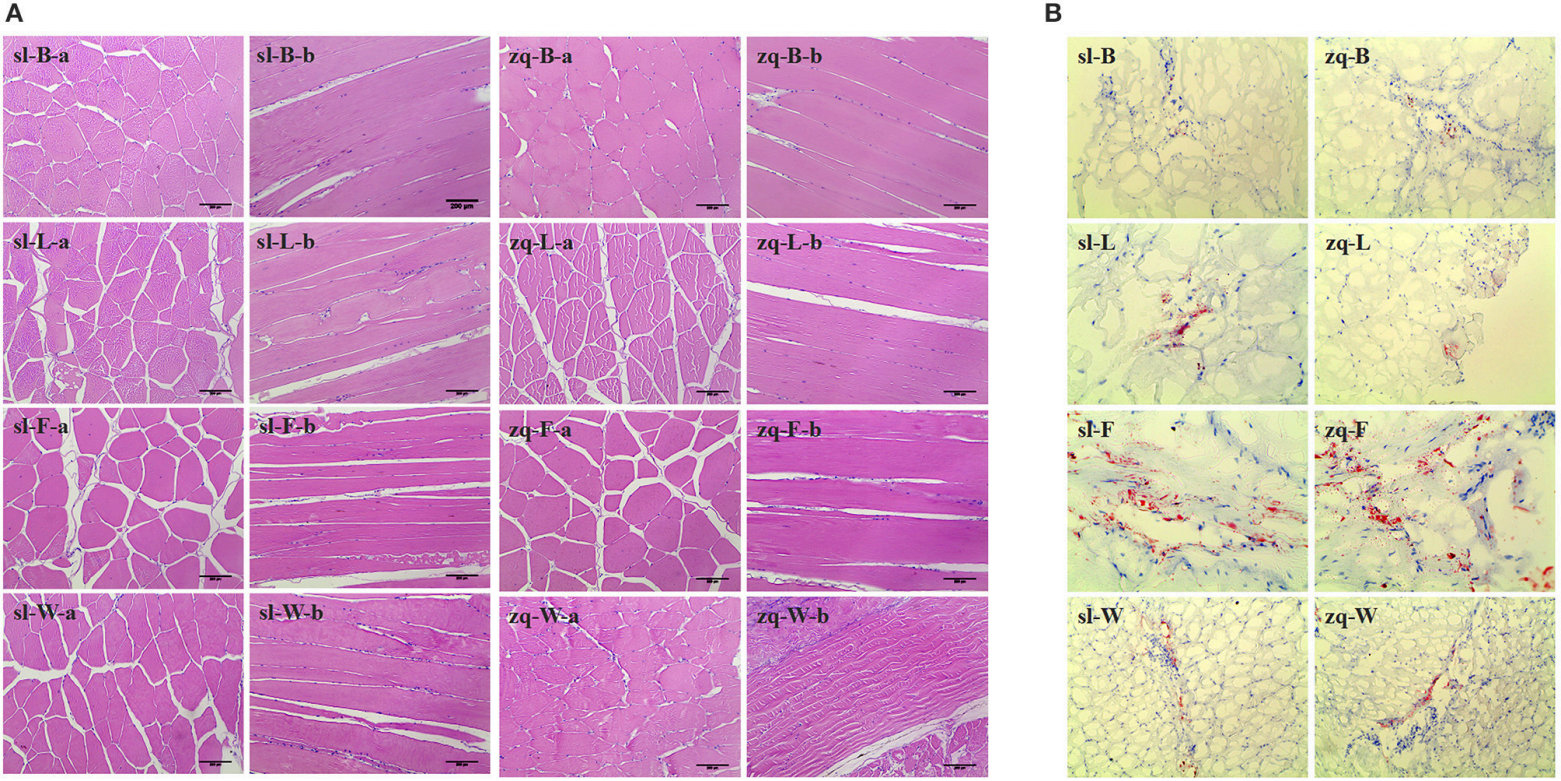
Tissue sections of different positional muscle of *C. idellus*. The different positional muscle samples were collected from artificial feed *C. idellus* (sl) and grass feed *C. idellus* (zq). Left part **(A)**, H&E staining (original magnification × 200) exhibits a distribution of muscle fiber and muscle fiber space. Right part **(B)**, oil red o staining (original magnification × 100) shows the intracytoplasmic fat (Red color). The capital letters B, L, F and W indicate namely-the back, rib, abdomen and tail muscles. In addition, lowercase letters—“a” and “b” (H&E staining) stand for the crosscutting and longitudinal cut respectively.

**Figure 4:**
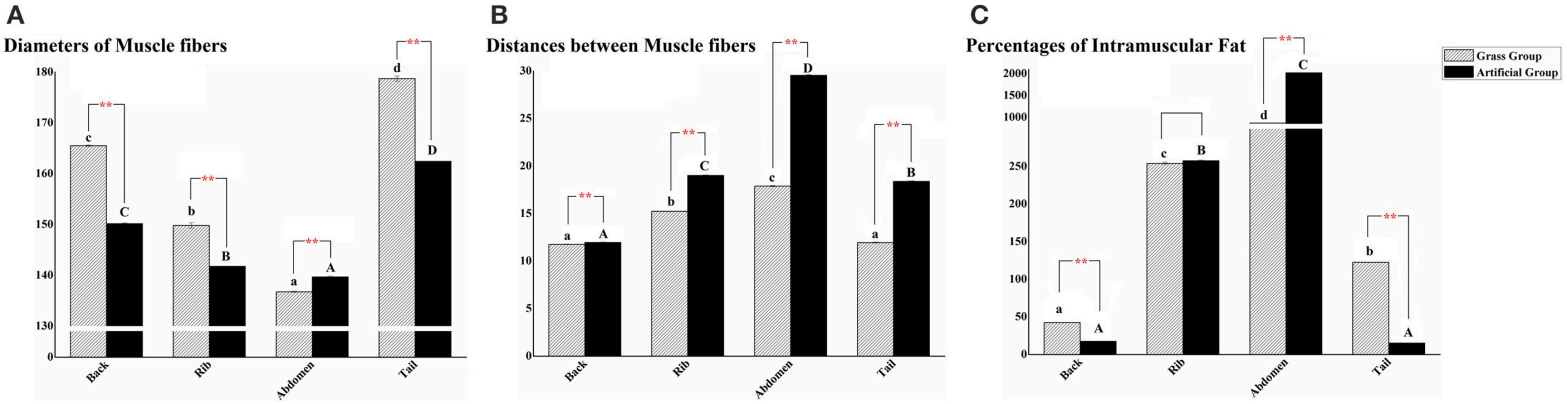
Quantitative results of different positional muscle tissue sections. Quantitative analysis of different positional muscle fiber sections of grass feed *C. idellus* (dense slash filling) and artificial feed *C. idellus* (black filling). The muscle fiber diameter statistics of four positional muscle tissues was shown in **(A)**. The comparisons of distances between muscle fibers and percentages of intramuscular lipid between the two groups were separately presented in **(B)** and **(C)**. Vertical bars represent the mean ± SE. The X axis represent back muscle, rib muscle, abdomen muscle, and tail muscle, respectively. Lowercase (grass feed group) and capital letters (artificial feed group) state the significant differences among the four positional muscle tissues sections (*P* < 0.05). While the great significance between the two models were marked with ^**^P < 0.01.

The histological changes were recorded using diameters of white muscle fibers, interstitium between myofibers, and filet lipid distributions (Figure 3). The average fiber diameters of back, rib, and tail muscle in AFG were significantly lower than those in GFG (*P* < 0.01), however, the opposite result occurred in abdomen muscle (Figure 4A). Compared with fish in GFG, the interspaces between myofibers of the four tested muscle parts were all evidently larger in AFG fish (*P* < 0.01; Figure 4B). There was no significant difference in fat content of rib muscle tissue between two groups. For back and tail muscle tissues, the filet lipid contents was significantly higher in GFG (*P* < 0.01). The trend is reversed for abdomen muscle tissue (Figure 4C).

### Gene expressions in various muscle tissues

The gene expression levels of *MRF*s varied either in nine positional white muscle tissues and one red muscle tissue in each group or in the same part of muscle between different groups. The expression levels of *MyoG* in various positional muscle samples, except for in tail muscle tissue, were 1.2–7.6 times higher than those in AFG (*P* < 0.01). *MyoD* expression levels were higher in back, rib, red, tail, paired fins, opercular, and eyes muscles of *C. idellus* in GFG. In the other muscular tissues, higher expression levels were observed in AFG (*P* < 0.01).

**Figure 5:**
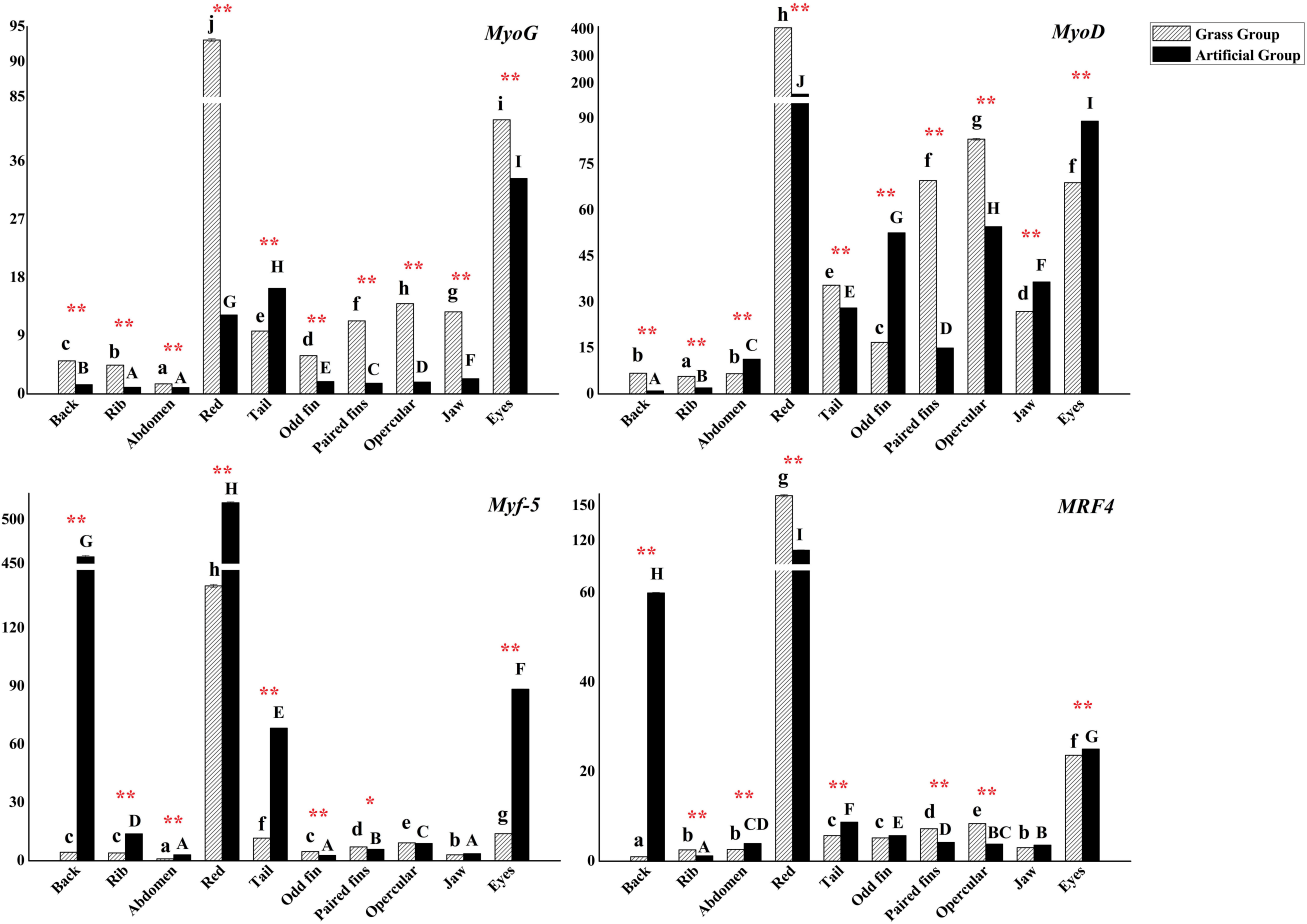
The expression levels of *C. idellus MyoG, MyoD, Myf*-*5*, and *MRF4* in 10 positional muscle tissues. The expression levels of *C. idellus MyoG, MyoD, Myf*-*5*, and *MRF4* in back, rib, abdomen, red, tail, odd fin, paired fins, opercular, jaw, and eyes muscle samples, which collected respectively from the two culture model *C. idellus*. The expressions of genes in grass feeding *C. idellus* (dense slash filling) and artificial feeds feeding *C. idellus* (black filling). The β-actin was used as an internal control to calibrate the cDNA template for all the samples. Vertical bars represent the mean ± SE. Different letters mean significantly different (*P* < 0.05) in the same model, lowercase letters used in grass feed group, capital letters used in artificial feed group. Meanwhile, the star bars also indicate the significance between the two culture models in red color, ^*^means significant (*P* < 0.05), ^**^means greatly significant (*P* < 0.01).

The expression patterns of *Mfy*-*5* and *MRF4* differed from *MyoG* and *MyoD*. These gene expressions were all influenced by both diet and sampling positions (*P* < 0.05). *Mfy*-*5* and *MRF4* expressed the most in red muscle of the two experimental groups. And their expression levels were significantly higher in back, eyes and tail muscle tissues of AFG, while higher expressions occurred in muscle tissues of eyes, tail, opercula, odd fin, and paired fins of fish from GFG (*P* < 0.05) (Figure 5).

**Figure 6:**
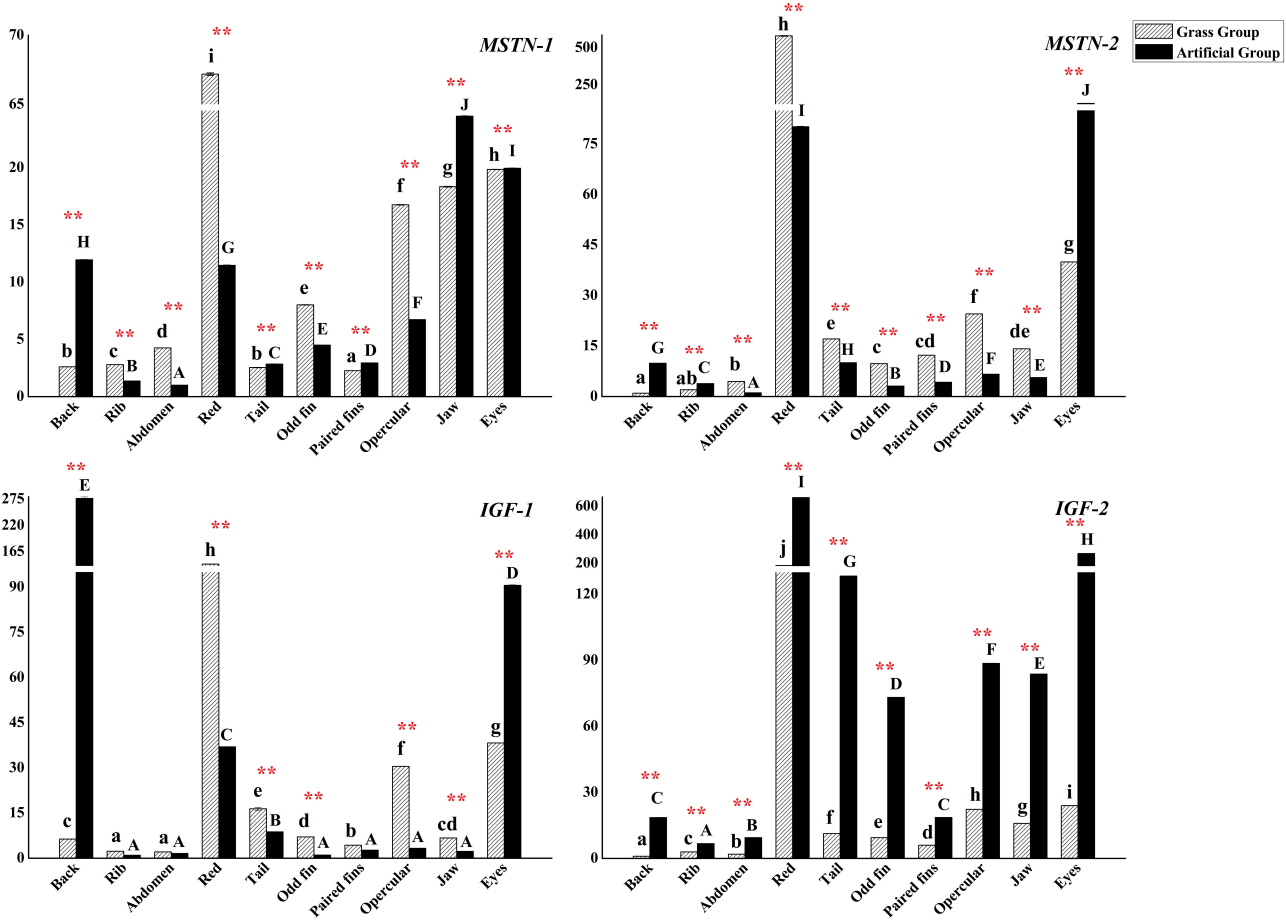
The expression levels of *C. idellus MSTN*-*1, MSTN*-*2, IGF*-*1*, and *IGF*-*2* in different muscle tissues. The expression levels of *C. idellus MSTN*-*1, MSTN*-*2, IGF*-*1*, and *IGF*-*2* in back, rib, abdomen, red, tail, odd fin, paired fins, opercular, jaw, and eyes positions of the two culture model *C. idellus*. The expressions of genes in grass feed group represented in dense slash filling, which in artificial feed represented in black filling. The β-actin was used as an internal control to calibrate the cDNA template for all the samples. Vertical bars represent the mean ± SE. Different letters mean significantly different (*P* < 0.05) in the same model, lowercase letters used in grass feed group, capital letters used in artificial feed group. Meanwhile, the star bars also indicate the significance between the two culture models in red color, ^*^ means significant (*P* < 0.05), ^**^ means greatly significant (*P* < 0.01).

Gene expressions of *MSTN*s (*MSTN*-*1* and *MSTN*-*2*) and *IGF*s (*IGF*-*1* and *IGF*-*2*) were plotted in Figure 6. The expression level of *MSTN*-*1* was higher in back, tail, paired fins, jaw, and eyes muscle samples of fish in AFG, and expressed lower in the other positional muscles (*P* < 0.0*1*). *MSTN*-*2* expressed much more in the most of sampled muscles in fish of GFG, except for lower expression level detected in the muscle of back, rib, and eyes (*P* < 0.01).

Gene expressions of *IGF*-1 and *IGF*-2 varied in the deferent sampling positions of muscle. *IGF*-*1* expressed significantly higher in GFG (*P* < 0.01), with a few exceptions of expressions in back and eyes muscles. Gene expression level of *IGF*-*2* was significantly higher in AFG (*P* < 0.01).

**Figure 7:**
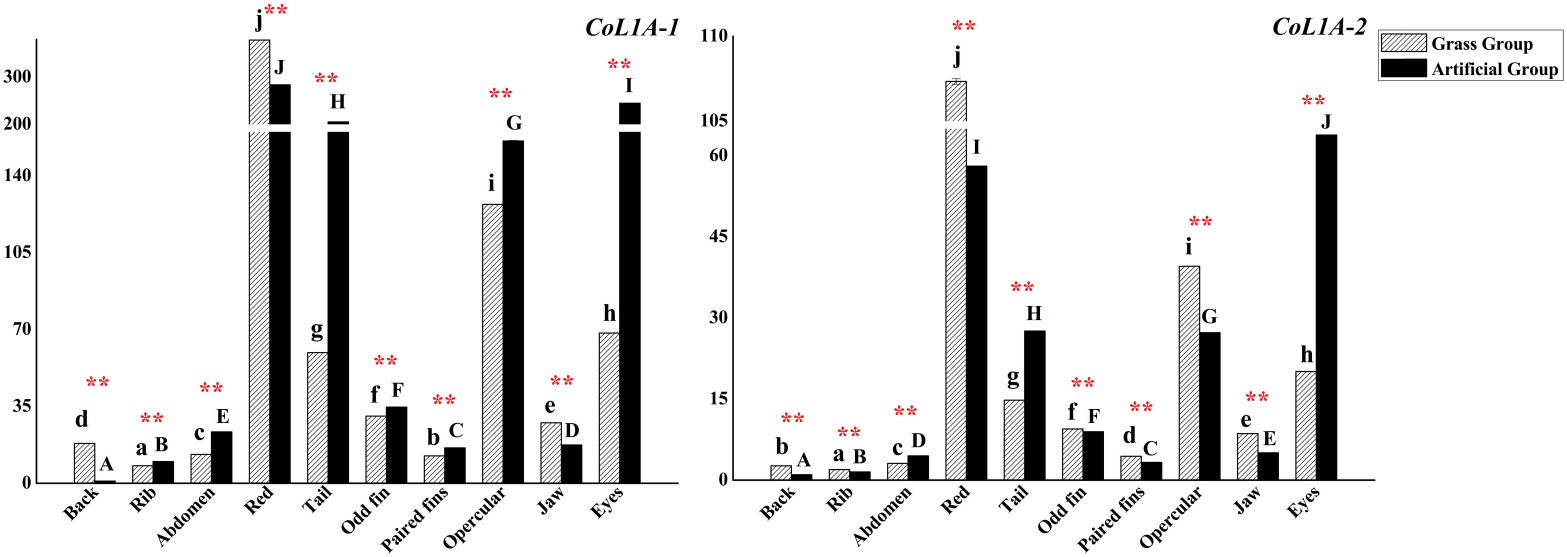
The expression levels of *C. idellus CoL1A*-*1* and *CoL1A*-*2* in different muscle tissues. The expression levels of *C. idellus CoL1A*-*1* and *CoL1A*-*2* in back, rib, abdomen, red, tail, odd fin, paired fins, opercular, jaw, and eyes positions of the two culture models *C. idellus*. The expressions of genes in grass feed group represented in dense slash filling, which in artificial feed represented in black filling. The β-actin was used as an internal control to calibrate the cDNA template for all the samples. Vertical bars represent the mean ± SE. Different letters mean significantly different (*P* < 0.05) in the same model, lowercase letters used in grass feed group, capital letters used in artificial feed group. Meanwhile, the star bars also indicate the significance between the two culture models in red color, ^**^means greatly significant (*P* < 0.01).

Collagen type I alpha were coded by two subtypes genes *CoL1A*-*1* and *CoL1A*-*2*, which had the similar expression patterns in two groups (Figure 7). The both genes had the highest expressions in red muscle and higher expressions in eyes muscle, opercular muscle, and tail muscle. *CoL1A*-*1* and *CoL1A*-*2* expressed lower in the muscle of back, rib, abdomen and paired fins. There was significant deference in the expressions of two genes in each sampled muscle tissue between groups (*P* < 0.01).

## Discussion

In present study, fish fed with succulence diets (*L. perenne, E. pectinata*, and *S. sudanense*) had a significantly slower growth performance than fish fed with artificial feed. It is consistent with previous observations, the negative correlation between the enhanced dietary fibers and the decreased weight gain, in *Barbodes altus* (Elangovan and Shim, 2002), *O. mykiss* (Harlioglu, 2011), even in *C. idellus* (Yu et al., 2014b; Cheng et al., 2016).

One potential caveat is that the growth performances and flesh quality could be affected by rearing conditions (Johnston, 2001; Yan et al., 2013; Gutierrez et al., 2014). However, in present study, temperature, DO and concentrations of various chemical elements were not significantly changed by feeding with different diets. Moreover, the variation trends of mineral element contents were similar in the two groups during the 4-month period. Only pH values were decreased by feeding with grass in the last 2 months. In summary, feeding with grass or artificial feed may cause the change of water indicators in aquaculture ponds under the same management conditions. Nevertheless, different diets are more important factor in the significant differences on growth traits and flesh quality, than the water environmental factors.

Serum biochemical parameters are often used as sensitive biomarker for confirming the aquatic product quality and for monitoring any changes in aquaculture in many fish species (Polakof et al., 2012). Many studies have shown that most serum parameters, such as ALP and cholesterol, are able to respond to the restricted nutrient intake, linking nutritional status with impaired weight status (Nova et al., 2008). In mammals, a study showed that in rats fed with fiber diets (celery, parsnip, and rutabaga), the fecal moisture content increased, body water content decreased, and serum biochemical parameters were also higher when compared with fiber-free control group (Mongeau et al., 1990). Similarly, the higher values of most parameters found in this study might also reflect lower moisture content in *C. idellus* fed with grass. Compared with AFG, significantly higher concentrations in LD, ALP, TCHO, HDLC, TP, and GLU content were found in GFG, *C. idellus* in GFG exhibited better physiological functions in response to the changes in food nutritional composition. This is consistent with results demonstrated in other fish species, e.g., *Pagrus major* (Mohammed et al., 2016) and *C. carpio* (Imanpoor et al., 2016). These findings suggest that feeding *C. idellus* with grass can promote the metabolism and transport of various substances in the body.

Flesh quality is affected by various intrinsic and extrinsic factors. The previous works proposed several potential factors, including breed, genotype, gender, diet, exercise, and ambient temperature, which can be used to manipulate muscle fiber characteristics and subsequently flesh quality in animals (Joo et al., 2013). Because the flesh texture depends primarily on muscle fiber characteristics, numerous studies have reported the relationship between quality traits and fiber characteristics (Johnston, 1999; Johnston et al., 2000). Texture also affects other characteristics of flesh quality such as appearance, flavor and nutrients of fish products (Anne et al., 2016). Generally, flesh quality involving in muscle texture and histological characteristics of *C. idellus* was affected by feeding regimes; for instance, feeding fish with different diets that made with varying amounts of nutrient compositions.

Muscular texture is governed primarily by the construction of muscle fibers and cytoplasm between myofibers (Taylor et al., 2002). High lipid content in filets has also been suggested as an important contributor to flesh soft texture (Bjørnevik et al., 2004). Changes in muscle fiber size can cause alteration in flesh texture. There are two closely associated factors affecting myofibrillar structure. One is rearing situations (e.g., culture conditions, storage times and feeding rations), the other is connective tissue, cross-linking between collagen molecules (Johnston et al., 2007; Fuentes et al., 2012). In this study, *C. Idellus* in AFG showed significantly higher filet lipid content in abdominal muscle tissues and larger distances between myofibers. Furthermore, fish muscle in AFG had lower values in texture parameters including hardness, resilience, and shear force. These results suggest that fish fed with artificial feed may exhibit more soft flesh traits. One important finding is that the fat deposition is most severe in abdominal muscle tissues collected from AFG, and feeding *C. idellus* with natural grass could effectively alleviate this problem.

The physiological processes of muscle growth, degradation and flesh quality are regulated by various molecular mechanisms (Salem et al., 2013). Since specific aspects of muscle growth and breakdown are dependent upon nutritional status, the expressions of genes involved in the myogenic signaling pathway are often analyzed to determine molecular response to different dietary (Johansen and Overturf, 2006). In this study, eight genes related to muscular growth and development, *MyoD, MyoG, Myf*-*5, MRF4, MSTN*-*1, MSTN*-*2, IGF*-*1*, and *IGF*-*2*, were quantitatively analyzed. These genes are the *C. idellus* 's homologs of the mammalian *MRF*s genes involved in myogenic signaling pathway and their positive and negative transcription factors (*IGF*s and *MSTN*) (Fuentes et al., 2013; Zeng et al., 2014). Slower growth rate was observed in GFG, coupled with significant decreased expressions of *Myf*-*5, MRF4*, and *IGF*-*2*. The expressions of *MyoD, MyoG, MSTN*s, and *CoL1A*-*2* were significantly upregulated in fish muscle in GFG. Similar results have been reported in many other fish species, including *O. mykiss* (Johansen and Overturf, 2006), *D. rerio* (Ulloa et al., 2013), *Solea senegalensis* (Luisa et al., 2016), and *C. idellus* (Yu et al., 2014a). *MyoG* levels always appear to be correlated with nutritional state in other organisms (Jeanplong et al., 2003). Our results suggest that grass feeding may lead to a rise in the amount of myotube proliferation and fusion, as well as significantly larger fiber diameters and numbers. Compared with AFG, the decreased transcription levels of *Myf*-*5, MRF4* as well as *IGF*-*2* in most of tested positional muscle tissues of GFG, could be induced by malnutrition.

*MSTN*-*1* and *MSTN*-*2* negatively regulate muscle growth of fish (Zheng et al., 2015). The elevated expressions of both genes in most of positional muscle tissues of GFG suggest that *MSTN*s may contribute to the decreased muscle growth of fish in GFG. Consistent with this notion, in a previous study of mammals, normal mice and cattle showed a dramatic decrease in skeletal muscle mass when compared with those carrying mutations in myostatin (Grobet et al., 1997; McPherron et al., 1997). Moreover, the expression levels of *MRF*s and their related regulatory genes had significant differences between the various tested positional muscle tissues, potentially due to the movement patterns and muscle activity of these tissues.

The relationship between the regulatory mechanism and texture variations has also been demonstrated in the research on the effect of feeding *C. idellus* with sole faba bean (*Vicia fab*a) on muscle firmness (Lin et al., 2009; Yu et al., 2014b). In this study, the diameter of muscle fiber increased in fish feed with grass, consistent with the increased expression of type I collagen (*CoL1A*-*1* and *CoL1A*-*2*) in grass feeding *C. idellus*. Although the regulatory mechanism of muscle firmness in *C. idellus* is still unclear, a few studies have identified some genes associated with texture variations of fish, e.g., *Salmo salar L*. (Larsson et al., 2012) and *C. idellus* (Yu et al., 2014b). The increased expression of *CoL1A*s may be one of the reasons why the fish fed with grass rather than artificial feed had much higher muscle firmness.

## Conclusion

Significant correlation was found between the difference in fish growth and diets. Differential expressions of *MRF*s, *IGF*s, and *MSTN*s genes may contribute to the changes in muscle growth and development of *C. idellus* fed with different diets. Elevated expressions of *MyoG* and *MyoD* also reflected higher potential of muscle regeneration in grass feeding group. Compared with the *C. idellus* feeding with artificial feed, the fish fed with grass showed overall more type I collagen content, increased muscle firmness, enhanced activity of muscle fiber hypertrophy, but declined growth rate. The significantly increased expressions of *CoL1A*-*1* and *CoL1A*-*2* in back muscles of *C. idellus* from GFG are positively correlated to stronger muscle firmness. Feeding grass can reduce fat deposits in fish abdomen.

## Author contributions

Most contributions to this research have been made by HZ, such as conception and design, acquisition of data, as well as analysis and interpretation of data, etc. DL, XH, and WC also participated in conception and design of this study. LL, XZ, and RT made contributions to samples collection. HZ and DL participate in drafting the manuscript and revising it critically for important intellectual content; DL and JX give final approval of the version to be submitted and any revised version.

### Conflict of interest statement

The authors declare that the research was conducted in the absence of any commercial or financial relationships that could be construed as a potential conflict of interest. The reviewer SL and handling Editor declared their shared affiliation.

## Footnotes

^1^NCBI. Available online at: http://www.Ncbi.Nlm.Nih.Gov/gorf/orfig.cgi (Accessed February 14, 2014).

^2^GeNorm. Available online: http://medgen.ugent.be/genorm/ (Accessed December 10, 2013).
